# Environmental and occupational risks to reproductive health in women service members and veterans

**DOI:** 10.3389/fpubh.2025.1628858

**Published:** 2025-08-13

**Authors:** Kendra L. Clark

**Affiliations:** ^1^Department of Obstetrics and Gynecology, Olson Center for Women’s Health, University of Nebraska Medical Center, Omaha, NE, United States; ^2^Department of Environmental, Agricultural, and Occupational Health, University of Nebraska Medical Center, Omaha, NE, United States; ^3^Department of Veterans Affairs Nebraska-Western Iowa Health Care System, Omaha, NE, United States

**Keywords:** environmental exposures, occupational exposures, women, military personnel, reproductive health

## Abstract

Women have played a vital role in the U.S. military for decades, with their presence steadily increasing. However, despite this growth, research on the unique occupational and environmental exposures they face remains limited, highlighting the need for greater understanding to improve reproductive health outcomes. Chemical exposures such as burn pit emissions, airborne particulates, heavy metals, and pesticides can disrupt hormone regulation and pose risks for fertility, miscarriage, preterm birth, and congenital anomalies. Additional risks include unsafe water sources, contaminated soil, increased vaccinations, and extreme environmental conditions. However, studies on these exposures remain inconsistent, with some indicating significant reproductive risks while others show minimal or no impact. This mini review highlights what is currently known about the impact of military-related environmental and occupational exposures on women’s reproductive health and identifies key gaps in the literature. Further research is essential to determine high-risk exposures, guide policy development, and support early intervention strategies. Addressing the long-term impact of military-related environmental exposures is crucial for ensuring better health outcomes and facilitating access to care for female service members and veterans.

## 1 Introduction

Women have been an integral part of the military for decades, serving across various roles and operational environments. As of 2023, women constitute nearly 18% of the active-duty force and approximately 11.3% of the total U.S. Veteran population (1), marking them as the fastest growing subgroup among Veterans. By 2043, women are projected to make up 17.2% of all living Veterans (1). As the presence of women in the armed forces continues to grow, so does the need to understand the unique occupational and environmental exposures they face during their time of service. While much research has examined the health effects of military service on men, fewer studies have focused on the specific exposures and risks encountered by women, particularly in relation to environmental and occupational hazards.

**Figure 1:**
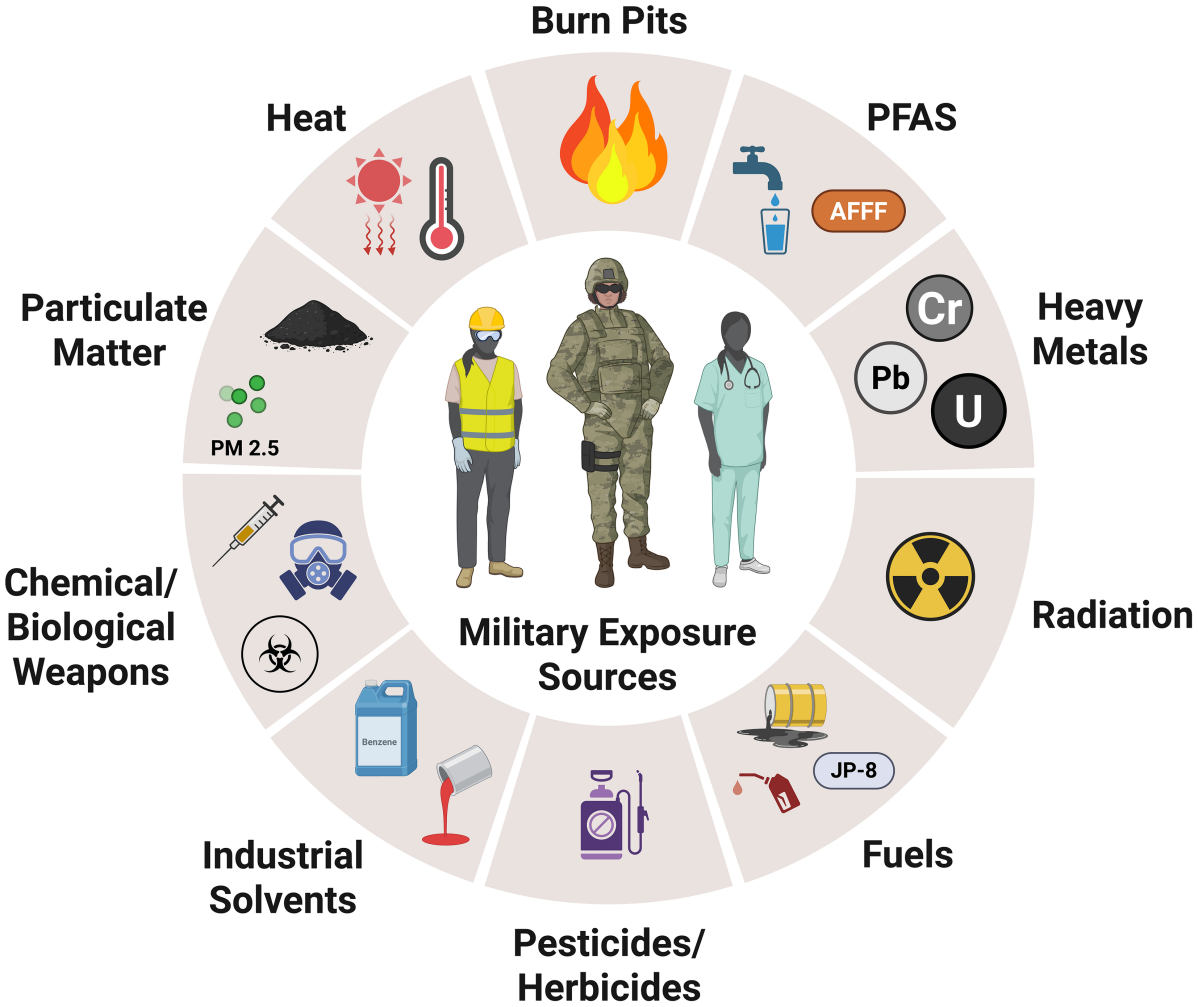
Potential occupational hazards, including chemical and physical exposures faced by female military personnel.

Military service inherently involves exposure to a variety of environmental and industrial hazards that can have lasting health consequences for both women and their offspring. Active-duty military women, who are often in their prime reproductive years, are particularly vulnerable to harmful chemical exposures that can disrupt hormone regulation and impair fertility (2–4). For women who become pregnant while serving, military exposures may pose increased risks for pregnancy complications including miscarriage, preterm birth, low birth weight, and congenital anomalies (4–8). These exposures include toxic substances such as burn pit emissions, airborne particulates, heavy metals, industrial solvents, fuel combustion byproducts, and pesticides (9–11) (Figure 1). Women serving in deployed settings may also encounter unsafe water sources, contaminated soil, increased routine and/or deployment-specific vaccinations, and prolonged exposure to extreme temperatures (9, 10), all of which may pose potential risks to reproductive health.

Understanding the lasting impact of military-related environmental exposures is essential for enhancing health outcomes and ensuring better access to care for servicewomen and veterans, especially during their transition to civilian life. As the Department of Veterans Affairs (VA) expands women-centered healthcare services (3, 12, 13), further research is needed to assess the reproductive effects of military service, identify high-risk exposures, and develop policies that mitigate potential health risks. Additionally, increasing awareness and education on these risks among both healthcare providers and service members will be essential in ensuring early intervention and targeted preventive measures. In this mini review, we examine studies on the impact of military environmental and occupational exposures on reproductive health outcomes in active-duty and veteran women (Table 1). Additionally, we identify gaps in understanding female-specific health concerns related to these exposures and highlight key areas for future research.

**Table 1:** Overview of main studies investigating exposures in female military personnel.

Author (year)	Exposure scenario	Study design	Population	Key findings
Exposure risks and reporting among military women				
Smith et al. (2007) (14)	Self-reported occupational exposures in deployed U.S. military women	Cross-sectional	10,539 active-duty women in the Millennium Cohort Study (2001–2003)	Certain military occupations (healthcare, electronic repair, electrical work, functional support roles) had higher odds of reporting chemical exposures
Carney et al. (2003) (15)	Self-reported combat-related and environmental exposures during Gulf War	Cross-sectional	129 deployed U.S. women compared to 1,767 deployed U.S. men from the Iowa Gulf War cohort (1990–1991)	Women reported similar combat exposures as men but greater post-deployment healthcare use
Lafferty et al. (2023) (16)	Self-reported Gulf War deployment exposures and chronic health issues	Qualitative	10 U.S. women Gulf War veterans (1990–1991)	Women faced similar deployment exposures as men; higher rates of reported chronic physical/mental health issues
Weaver et al. (2024) (17)	Exposure to contaminated drinking water at Camp Lejeune	Retrospective cohort	U.S. Women Veteran Marines stationed at Camp Lejeune (4,491) and Camp Pendleton (2,811) between 1975 and 1985 and utilized VA healthcare between 1999 and 2001	No direct link between toxicant exposure and overall disability rating; exposure associated with presumptive conditions only when comparing bases
Stuart et al. (2003) (18)	Self-reported belief in exposure to nerve/mustard gas among U.S. Gulf War veterans	Retrospective cohort	4,620 female Gulf War veterans (subset of 44,168 total Department of Defense Gulf War Registry participants 1994–1998)	Women more likely than men to believe they were exposed to chemical agents; belief associated with more symptoms, poorer health, and higher rates of mental health diagnoses
Deployment and fertility challenges				
Ippolito et al. (2016) (19)	Self-reported infertility and miscarriage incidence in deployed U.S. military women	Prospective cohort	11,183 previously deployed female veterans for impaired fecundity analysis; 3,366 included in miscarriage analysis (Millennium Cohort Study survey years 2004–2006 and 2007–2008)	Military deployment did not result in increased rates of impaired fecundity or miscarriage
Katon et al. (2014) (20)	Self-reported infertility in U.S. veteran men and women	Retrospective cohort	20,370 OEF/OIF veterans (4,314 women, 16,056 men) from National Health Study for a New Generation of U.S. Veterans (2009–2011)	Female veterans reported similar rates of infertility (15.8%) vs. males (13.8%)
Mancuso et al. (2020) (21)	Infertility rates and perceived quality of life in U.S. women veterans	Cross-sectional	996 women veterans enrolled in Women Veterans Reproductive Health Study (2000–2008)	~18% of women veterans reported infertility and had lower perceived physical wellbeing
Goossen et al. (2019) (22)	Race/ethnicity and self-reported infertility, care access, and treatment	Cross-sectional	1,004 U.S. Midwestern VA-enrolled women; 802 white race, 202 racial minority	Racial minority veterans more likely to report infertility, but less likely to receive treatment
Mattocks et al. (2015) (23)	VA utilization and infertility diagnosis/treatment among U.S. OEF/OIF/OND veterans	Retrospective cohort	68,442 OEF/OIF/OND women veterans using VA care (Defense Manpower Data Center Contingency Tracking System Deployment file 2001–2010)	~2% received infertility diagnosis; higher rates among Black, Hispanic, obese
Military environmental exposures and fertility outcomes				
Reutman et al. (2002) (24)	Jet fuel handling and solvent exposures	Cross-sectional	100 female USAF employees	Military occupation with solvent/fuel exposure linked to lower LH and progesterone production
Gordley et al. (2000) (25)	Jet fuel handling	Cross-sectional	170 U.S. military and civilian women from 10 USAF bases	Military occupation with fuel exposures reported higher incidence of menstrual disorders
Mancuso et al. (2022) (26)	Self-reported occupational exposures in U.S. women veterans	Cross-sectional	1,194 women identified from random sampling of the US Department of VA and Department of Defense Information Repository (VADIR)	Women veterans experiencing infertility had higher/more frequent reported occupational chemical exposures than women veterans not experiencing infertility
Altman et al. (2024) (27)	Military environmental exposures during Gulf War	Prospective cohort	2,540 women U.S. veterans (Gulf War cohort 1995–2012)	Exposures associated with increased odds of infertility
Jacobson et al. (2008) (28)	Smallpox vaccination among active-duty U.S. service members	Retrospective cohort	44,332 active-duty women (12,519 smallpox vaccinated, 31,813 unvaccinated with active-duty service from 2003 to 2005)	No significant association between smallpox vaccination and infertility diagnosis
Pregnancy, birth outcomes, and deployment				
Ryan et al. (2011) (29)	Military service and deployment-related exposures	Retrospective cohort	63,056 infants born to U.S. military women (22,596 born to women with deployment experience before/during/after pregnancy, 40,460 born to non-deployed military women) from US DOD Birth and Infant Health Registry 2002–2005	Reported no significant adverse birth outcomes related to deployed versus non-deployed servicewomen
Araneta et al. (2000) (30)	Gulf War deployment and exposures	Retrospective cohort	1,265 infants born to female U.S. military personnel (165 Gulf War veteran infants, 1,100 non-deployed veteran infants born in Hawaii between 1989 and 1993)	No specific birth defects in infants of Gulf War deployed women versus non-deployed servicewomen
Kang et al. (2000) (31)	Vietnam War service	Retrospective cohort	8,280 Vietnam War-era female veterans (4,140 women Vietnam veterans, 4,140 non-Vietnam veterans with service between 1965 and 1973)	Increased reports of moderate-to-severe birth defects among infants of deployed veterans
Shaw et al. (2018) (32)	Military service and deployment	Retrospective cohort	12,877 infants born to U.S. servicewomen during periods of active duty between 2011 and 2014	Deployment timing associated with increased risk of adverse pregnancy outcomes
Kang et al. (2001) (33)	Gulf War service	Retrospective cohort	1,331 Gulf War-era female U.S. veterans with index pregnancy	Increased reports of moderate-to-severe birth defects among infants of deployed veterans; more reported miscarriages and stillbirths
Araneta et al. (2004) (34)	Gulf War deployment	Retrospective cohort	1,558 Gulf War-era female U.S. veterans pregnant between 1990 and 1992	Higher risk of spontaneous abortions and ectopic pregnancy in post-Gulf War conceptions
Katon et al. (2014) (35)	OEF/OIF deployment and pregnancy	Retrospective cohort	2,288 OEF/OIF women U.S. veterans who used VA maternity benefits between 2001 and 2010	OEF/OIF veterans had higher risk of developing GDM than stateside female veterans
Araneta et al. (2003) (36)	Gulf War deployment	Retrospective cohort	4,416 Gulf War-era female U.S. veterans (450 women Gulf War veterans, 3,966 non-deployed veterans who gave birth between 1989 and 1993)	Higher risk of specific birth defects (hypospadias) in male infants of previously deployed female Gulf War veterans versus non-deployed women
Bukowinski et al. (2017) (37)	Military service and deployment	Retrospective cohort	174,921 infants born to U.S. active-duty women between 2003 and 2014 (DOD Birth and Infant Health Registry)	Rates of birth defects, preterm birth, and low birth weight similar or lower than general US population; increased risk of hypospadias in male infants
Military environmental exposures and pregnancy/birth outcomes				
Magann et al. (2009) (38)	Military service and pregnancy	Prospective cohort	814 active-duty U.S. military women with low-risk pregnancies	Noise exposure and prolonged standing increased risk of preterm birth
Stark et al. (2024) (39)	Military aviation exposure	Retrospective cohort	25,929 pregnant U.S. active-duty female officers with 46, 323 total pregnancies (2,131 aviation officer pregnancies, 44,192 non-aviation officer pregnancies between 2002 and 2019)	Increased risk of placental complications and fetal growth restriction, decreased risk for gestational diabetes and hypertension
Stark et al. (2024) (40)	Military aviation exposure	Retrospective cohort	27,033 pregnant U.S. active-duty female officers (1,144 infants born to aviation officers, 25,889 infants born to non-aviation officers between 2002 and 2019)	Decreased risk of neonatal growth abnormalities and adverse neonatal health outcomes
Wiesen et al. (2002) (41)	Pre-pregnancy Anthrax vaccination	Retrospective cohort	U.S. Army women (17–44 years old, 3,136 anthrax vaccinated, 962 unvaccinated stationed in Georgia between 1999 and 2000)	No impact on pregnancy, birth rate, or adverse birth outcomes
Ryan et al. (2008) (42)	Anthrax vaccination during pregnancy	Retrospective cohort	115,169 infants born to 95,595 U.S. active-duty women between 1998 and 2004	Slight increase in birth defects of first trimester exposed infants
Ryan et al. (2008) (43)	Smallpox vaccine during pregnancy	Retrospective cohort	31,420 infants born to U.S. active-duty military women between 2003 and 2004	No significant association of smallpox vaccination with preterm birth or birth defects
Conlin et al. (2013) (44)	Pandemic H1N1 vaccination during pregnancy	Retrospective cohort	10,376 active-duty U.S. military women who received H1N1 vaccination during pregnancy between 2009 and 2010; 7,560 active-duty U.S. military women who received seasonal influenza vaccination during pregnancy between 2008 and 2009	No significant differences in adverse outcomes between H1N1 and seasonal flu vaccine groups
Friedman et al. (2022) (45)	Gulf War deployment	Retrospective cohort	239 women U.S. veterans from the Gulf War Women’s Cohort study who served between 1990 and 1991	High prevalence of adverse reproductive outcomes including infertility, miscarriage, stillbirth, and pregnancy complications; pesticide use associated with increased odds of outcomes
Warner et al. (2022) (46)	Iraq and Afghanistan deployment	Retrospective cohort	1,730 Australian Defense Force women; Middle East Area of Operations (MEAO) Census study 2001–2009	Women who deployed to both Iraq and Afghanistan had significantly more adverse reproductive outcomes
Hourani & Hilton (2000) (47)	Self-reported occupational exposures in U.S. Navy women	Case-control	1,032 U.S. Navy women pregnant in 1993	Active-duty women with reported exposures were more likely to experience preterm labor
Bukowinski et al. (2012) (48)	Gulf War deployment and exposures	Retrospective cohort	26,617 infants born between 1998 and 2004 to Gulf War-era U.S. military women	No overall increase in birth defects for infants of deployed vs. non-deployed veterans
Conlin et al. (2012) (49)	Burn pit exposure during deployment to Iraq and Afghanistan	Retrospective cohort	13,129 infants born between 2004 and 2007 to U.S. active-duty women	No overall increase in risk in birth defects, male infants born to USAF mothers more likely to be born with birth defect

## 2 Military service and reproductive health: risks, exposures, and outcomes

### 2.1 Exposure risks and reporting among military women

Several studies have highlighted significant gender differences in exposure reporting and risks, perceived health impacts, and post-exposure healthcare usage. Thus, the accurate assessment of these exposures is crucial for understanding long-term health risks among female service members. Using data from women in the Millennium Cohort Study, researchers found that both active-duty and Reserve/National Guard women in occupations such as healthcare, electronic repair/electrical work, and functional support roles faced the highest exposure risks (14). These women frequently reported encounters with death, trauma, chemical and biological warfare agents, depleted uranium, and pesticides, highlighting the occupational hazards associated with these roles (14). Active-duty women, particularly those in the Army, experienced greater exposure risks compared to their counterparts in the Reserve or National Guard (14). Other studies of women who served during the Gulf War (GW) indicated that women and men reported similar rates of overall environmental exposures (15, 16). Women, however, were more likely to experience illness at the time of exposure, particularly those related to smoke from oil fires, burning trash/human waste, mustard gas, or food contaminated with smoke, oil, or chemical agents (15). Additional exposures reported by a smaller cohort of GW women veterans included extreme heat and sand, oil fires/burn pits, depleted uranium, chemical munitions, vaccinations, and pyridostigmine bromide pills (16). Notably, 5 years post-deployment, women demonstrated higher rates of both outpatient and inpatient healthcare utilization compared to men and were more likely to receive disability compensation from the VA (15). Among female veterans stationed at Marine Corps Base Camp Lejeune, where water contamination raised concerns about long-term health risks, a comparative study with women stationed at Camp Pendleton, an unexposed control site, found no direct association between toxicant exposure and higher disability ratings (17). However, women from Camp Lejeune were more likely to have service-connected disability claims for presumptive conditions linked to toxicant exposures (17). Additionally, reproductive health concerns, such as ovary removal, were reported more frequently among exposed women (17). Beyond documented exposures, belief in toxicant exposure also may influence health outcomes. Women GW veterans were more likely to report perceived exposure to nerve or mustard gas and reported greater encounters with other potentially toxic agents and traumatic combat experiences than male GW veterans (18). Further, such beliefs resulted in higher rates of physical symptoms, mental health diagnoses, and poorer self-reported health in GW women veterans (16, 18). These findings suggest that the psychological impact of perceived exposure, regardless of documented exposure status, may contribute to increased healthcare utilization and long-term illness risk. Addressing both real and perceived exposures in military healthcare settings is essential for improving health outcomes and reducing the burden of post-deployment illnesses.

### 2.2 Deployment and fertility challenges

The impact of military deployment on fertility remains a growing area of concern, particularly as more women serve. Another study using data from the Millennium Cohort Study examined whether deployment-related experiences, including combat exposure and stress, were associated with an increased risk of impaired fecundity among U.S. servicewomen. Their findings indicated that military deployment, regardless of combat exposure, was not significantly associated with increased rates of infertility or miscarriage (19). Similarly, a study that examined self-reported infertility among veterans who served during Operation Enduring Freedom/Operation Iraqi Freedom (OEI/OIF) found that while female veterans were no more likely than male veterans to report infertility, they were significantly more likely to seek medical help for fertility issues (20). Alternatively, survey data from VA-enrolled women aged 21–52 found that 18% reported a history of infertility, and those with a history of infertility reported lower perceived physical wellbeing and higher rates of chronic conditions such as fibromyalgia, persistent pain, and cancer (21). Further, racial minority veterans were more likely to report infertility but less likely to receive infertility treatment compared to their white counterparts (22, 23), citing reasons for not seeking fertility treatment including lack of awareness about available evaluations and treatments, as well as uncertainty or mixed feelings about becoming pregnant (22). While these studies suggests that military service may not significantly alter infertility prevalence compared to civilians, the demands of deployment may delay fertility. Further, these findings suggest that infertility may serve as an indicator of broader health concerns, which is likely to result in greater healthcare utilization for reproductive issues. However, given the limited scope and timespan of the data sets evaluated in the aforementioned studies, further research is needed to fully understand the long-term reproductive effects of military service, given that more women are currently serving in deployment situations than ever before.

### 2.3 Military environmental exposures and fertility outcomes

A small number of studies have explored the associations between chemical exposures, hazardous materials, and reproductive endocrine disruptions among female service members and veterans. Fuel handling is a common occupational exposure in the military, and a small number of studies have examined its impact on reproductive health among military women. Research on U.S. Air Force personnel exposed to jet fuel (JP-8) and organic solvents found that women with higher exposure to aliphatic (C_6_H_14_–C_16_H_34_) and aromatic (benzene, ethylbenzene, toluene, *m,p,o*-xylenes) hydrocarbons exhibited lower preovulatory luteinizing hormone (LH) levels, a key hormone for ovulation and fertility (24). Additionally, exposure to aromatic hydrocarbons was associated with a trend toward lower midluteal progesterone levels, further suggesting potential endocrine disruption (24). Another study on female U.S. Air Force personnel who reported occupational fuel handling, found an increased risk of menstrual disorders such as dysmenorrhea and abnormal cycle lengths (25). Further supporting the connection between military toxicant exposure and reproductive health, another study found that women experiencing infertility had higher exposure rates to petrochemicals, polychlorinated biphenyls (PCBs), sulfur fires, extreme heat, and receiving an anthrax vaccine compared to those not experiencing infertility (26). Women with infertility also reported a greater cumulative number of toxicant exposures (26), suggesting that the burden of multiple exposures may compound reproductive risks. Similarly, research on female GW veterans using data from the Gulf War Longitudinal Cohort Study found that deployment, environmental exposures, and Gulf War Illness (GWI) were associated with an increased likelihood of infertility (27). Finally, military service members undergo various vaccinations as part of their duty requirements, raising questions about potential impacts on reproductive health, including infertility. A study of active-duty U.S. military women who received a smallpox vaccine determined that while the proportion of infertility diagnosis was slightly higher in vaccinated versus unvaccinated women, these differences were not statistically significant, indicative of no causal relationship (28). Taken together, these studies highlight the potential reproductive risks associated with military occupational and environmental exposures, particularly fuel handling, chemical mixtures, and deployment-related toxicants and vaccinations. Given the cumulative impact of multiple exposures, further research is needed to fully understand the long-term reproductive consequences and to develop targeted healthcare interventions for military women.

### 2.4 Pregnancy, birth outcomes, and deployment

Research on deployment and its effects on pregnancy and birth outcomes has yielded mixed results. Pregnancies among women who deployed during early pregnancy showed no significant increase in preterm birth or birth defects compared to non-deployed servicewomen (29). Similarly, an analysis of birth defects surveillance records revealed no significant differences in the prevalence of 48 selected birth defects between infants of female GW veterans and those of non-deployed female veterans (30). Furthermore, birth defect rates were consistent among infants conceived both before and after deployment, indicating no clear link between GW service and an increased risk of congenital anomalies (30). Likewise, among women Vietnam veterans, military service in Vietnam was not linked to an increased risk of miscarriage, stillbirth, low birth weight, preterm birth, or infant death, however there was an increased risk of birth defects (31).

In contrast, other research has identified greater risks of pregnancy complications, and birth defects associated with military service. Deployment timing may play a role, as women who gave birth within 6 months of returning from deployment had an increased risk of spontaneous preterm birth compared to those who had not recently deployed (32). Female GW veterans also reported more miscarriages and stillbirths than non-deployed counterparts, though these outcomes were found not statistically significant (33). Spontaneous abortions and ectopic pregnancies were also elevated in post-GW conceptions (34). Additionally, rates of gestational diabetes during pregnancy were higher in women veterans that were deployed in OEF/OIF versus women who were stateside (35). Concerns about birth defects among children born to military women have also been raised. An increased prevalence of certain congenital anomalies, particularly hypospadias, was observed among male infants conceived post-deployment by female GW veterans (36). Similarly, another study identified hypospadias as the most common birth defect reported in male infants born to active-duty mothers (37). Additionally, another study of female GW veterans found a higher prevalence of birth defects among children born to deployed female GW veterans versus non-deployed female GW veterans (33). Overall, while some studies suggest minimal pregnancy and birth risks associated with military service and deployment, others highlight specific vulnerabilities that warrant further investigation. Continued research is necessary to identify modifiable risk factors, improve obstetric healthcare for military women, and ensure comprehensive monitoring of birth outcomes in female service members and veterans.

### 2.5 Military environmental exposures and pregnancy/birth outcomes

Military women face a range of occupational and environmental hazards that could impact pregnancy and birth outcomes, with factors such physical demands and service-related exposures potentially contributing to increased pregnancy risks. The effects of standing, lifting, and noise exposure among active-duty pregnant women were examined, revealing that prolonged standing and noise exposure was associated with an increased risk of preterm labor/birth (38). The impact of aviation-related military careers on pregnancy has also been studied, showing that while military aviation officers had a lower risk of gestational diabetes and hypertension, they faced an increased risk of placental complications and fetal growth restriction, potentially due to occupational stressors such as intermittent hypoxia, circadian disruption, excessive noise/vibration, and radiation exposure (39). Interestingly though, babies born to female aviation officers were reported to less likely experience neonatal growth abnormalities or adverse neonatal health outcomes such as cardiovascular, neurological, or pulmonary disorders (40).

Concerns about the potential reproductive effects of vaccination in military women have led to several studies evaluating their impact on pregnancy and birth outcomes. The relationship between pre-pregnancy anthrax vaccination and pregnancy outcomes was examined in a study analyzing pregnancy rates, birth rates, and adverse birth outcomes among U.S. Army women. Findings showed no significant differences in these outcomes between vaccinated and unvaccinated women, though the study lacked sufficient power to detect rare adverse effects (41). In contrast, an analysis of birth defects among infants born to military women who received anthrax vaccinations found a slight increase in birth defects among first trimester-exposed infants, but the association was not statistically significant (42). The impact of smallpox vaccination on preterm births and birth defects among infants born to military women has also been examined, with findings showing no significant association between maternal smallpox vaccination and an increased risk of these outcomes (43). Another study assessed the safety of the pandemic H1N1 vaccine among pregnant military women and their newborns. The analysis compared pregnancy loss, preeclampsia, preterm labor, preterm birth, birth defects, and fetal growth problems between those vaccinated with the H1N1 vaccine (2009–2010) and those who received the seasonal influenza vaccine (2008–2009), with no significant differences in these outcomes between the two groups (44). Overall, studies on vaccine exposures in military women provide reassuring evidence that anthrax, smallpox, and influenza vaccinations do not significantly impact pregnancy outcomes or increase the risk of birth defects. Given the critical role of vaccination in force readiness and protection against biological threats, these findings support continued immunization efforts while emphasizing the need for ongoing surveillance to monitor any potential long-term reproductive effects.

Similar to the aforementioned studies on deployment-related environmental exposures and fertility outcomes, the role of exposures on birth outcomes have also yielded conflicting results. Higher rates of miscarriage, stillbirth, and pregnancy complications have been reported among GW women veterans, with pesticide cream use during deployment increasing the likelihood of women experiencing these outcomes (45). Similarly, Australian Defense Force veterans who deployed to both Iraq and Afghanistan and reported exposure to reproductive toxicants including exhaust emissions, fumes, toxic industrial chemicals, noise, and radiation also reported higher rates of adverse pregnancy outcomes including miscarriage, birth defects, and ectopic pregnancies (46). Active-duty U.S. Navy women were more likely to report exposures to heavy metals, solvents, and petroleum products at work after their pregnancies were confirmed relative to their civilian counterparts (47). Further, they are more likely to experience preterm labor during pregnancy (47). Alternatively, no significant increase in birth defects was observed among infants born to 1990–1991 GW female veterans, either at birth or within the first year of life (48). Research on burn pit exposure has also been inconclusive, with findings showing no consistent association between burn pit exposure including deployment location relative to the burn pit, timing, and duration of exposure before pregnancy and an increased risk of birth defects or preterm birth (49). Interestingly though, the male infants born to active-duty military women were more likely to be diagnosed with a birth defect if their mother was in the Air Force (49), potentially due to differences in occupational exposure scenarios. These findings underscore the complex relationship between military occupational and environmental exposures and pregnancy outcomes. While certain exposures, such as prolonged standing, aviation-related stressors, and deployment toxicants have been linked to increased offspring health risks, research on vaccine safety and birth defect prevalence has provided reassuring results. Continued investigation is necessary to clarify conflicting findings and ensure comprehensive reproductive healthcare for military women.

## 3 Conclusions and future perspective

As the number of women in active-duty military roles continues to grow, it is increasingly important to address their unique reproductive health risks. This review highlights the complex relationship between military related occupational and environmental exposures and reproductive health outcomes among female service members and veterans. While military service provides unique career opportunities for women, it also presents distinct risks to reproductive health through exposure to hazardous chemicals, high physical demands, and deployment-related environmental conditions. Despite growing recognition of these risks, research on their long-term reproductive impacts remains limited. Inconsistencies across studies point to a need for more robust data to establish clearer causal links between military toxicant exposures and adverse reproductive outcomes, emphasizing the importance of comprehensive exposure assessments and longitudinal studies. Moreover, disparities in infertility diagnosis and access to reproductive care persist within the military healthcare system, underscoring the importance of addressing systemic barriers to reproductive health services for servicewomen and veterans. Ultimately, supporting the reproductive health of women in the military requires a stronger focus on research, improved healthcare access, and a deeper understanding of the effects of military-related exposure and their risks.
